# Molecular Pathology of Cardiomyopathies: Bridging Morphology, Genomics, and Clinical Phenotypes

**DOI:** 10.3390/cimb48010060

**Published:** 2026-01-05

**Authors:** Andrea Marzullo, Cecilia Salzillo

**Affiliations:** 1 Pathology Unit, Department of Precision and Regenerative Medicine and Ionian Area, University of Bari Aldo Moro, 70124 Bari, Italy; 2 PhD Course in Public Health, Department of Experimental Medicine, University of Campania Luigi Vanvitelli, 80138 Naples, Italy

**Keywords:** cardiomyopathy, molecular pathology, genotype-phenotype correlation, sudden cardiac death, genetic testing, myocardial disease

## Abstract

Cardiomyopathies represent a heterogeneous group of myocardial diseases that share overlapping clinical and genetic profiles but distinct morphological and molecular signatures. Advances in molecular genetics and next-generation sequencing have revolutionized the diagnostic landscape, revealing that up to 60% of cardiomyopathies have an identifiable genetic basis. From a pathologist’s perspective, integrating histopathological findings with molecular data is crucial for understanding genotype–phenotype correlations and for guiding precision medicine. This review provides an updated overview of the molecular pathology of major cardiomyopathy subtypes, including dilated, hypertrophic, restrictive, arrhythmogenic, and non-compaction forms. For each entity, we discuss morphologic hallmarks, genetic mechanisms, and their impact on disease progression and sudden cardiac death. Special emphasis is placed on the role of desmosomal, sarcomeric, and cytoskeletal proteins in myocardial structure and function, and on how their mutations disrupt cardiomyocyte integrity and signaling pathways. Furthermore, we address the emerging role of molecular autopsy in unexplained sudden cardiac death, underscoring the importance of multidisciplinary collaboration among pathologists, geneticists, and clinicians. Finally, we highlight future directions in molecular diagnostics and targeted therapies, which are reshaping the classification and management of cardiomyopathies.

## 1. Introduction

Cardiomyopathies are a heterogeneous group of primary heart muscle diseases, characterized by structural and functional alterations of the myocardium in the absence of pathologies (coronary, valvular, or congenital) that fully explain the clinical picture. These diseases include dilated cardiomyopathy (DCM), hypertrophic cardiomyopathy (HCM), restrictive cardiomyopathy (RCM), arrhythmogenic cardiomyopathy (ACM) and left ventricular non-compaction (LVNC), manifesting with a wide clinical range from heart failure to arrhythmias, up to sudden cardiac death (SCD) [1,2].

Epidemiologically, inherited cardiomyopathies show variable prevalence. HCM is estimated to occur in approximately 1 in 500 individuals in the general population [3]. DCM has an estimated prevalence of around 1 in 2500 in the general population [4]. European and U.S. studies suggest that these estimates are perhaps conservative, given that many carriers of pathogenic genetic variants do not develop a fully manifest phenotype [4].

An analysis of a medical record-based cohort showed that the recorded prevalence of cardiomyopathies increased over time; for example, between 2010 and 2018, the prevalence of HCM increased by 9% and that of ACM by 180% [5].

Other epidemiological data come from genetics. A study of over 200,000 UK Biobank participants who underwent whole-exome sequencing (WES) found “pathogenic” or “likely pathogenic” variants associated with DCM, HCM and ARVC with a prevalence of approximately 1 in 251 for DCM, 1 in 149 for HCM, and 1 in 578 for ARVC. Despite the high presence of these variants in the population, phenotypic penetration often remains low, between 1.2% and 3.1%, which highlights a strong heterogeneity in disease expression [6].

Integrated diagnosis, which combines morphological data, obtained through imaging such as echocardiography or magnetic resonance imaging and, in appropriate cases, histological examination, with molecular-genetic data, has become crucial. This multidisciplinary approach improves diagnostic accuracy, prognostic stratification and therapeutic management, as well as family counseling [7].

Genetics plays an increasingly important role. Next-generation sequencing (NGS) technologies allow for the analysis of multigene panels, the WES or whole-genome sequencing (WGS), facilitating the identification of pathogenic variants. Furthermore, molecular autopsy (MA) on post-mortem samples has proven to be a fundamental tool in cases of sudden unexplained death (SUD), as it can reveal responsible genetic mutations that escape pathological analysis alone [7].

The aim of this literature review is twofold: to provide an updated overview of the state-of-the-art in the morphological and molecular assessment of cardiomyopathies, and to discuss the clinical and forensic implications of genetics in diagnosis, with particular attention to the integration of histological, genomic, and clinical data. The underlying rationale is that a “precise” diagnosis can significantly improve risk stratification, therapeutic management, and prevention, including in the patients’ families.

## 2. Cardiomyopathy Classification

Cardiomyopathies are commonly grouped into several major phenotypic categories, which include dilated DCM, HCM, RCM, ACM, often referred to as arrhythmogenic right ventricular cardiomyopathy (ARVC), and LVNC. These distinctions arise from morphological, functional and, increasingly, genetic differences, but do not represent rigidly separate compartments; in fact, numerous overlaps and intermediate variants exist [2,8].

The classification of cardiomyopathies has evolved considerably over time, reflecting advances in diagnostic techniques such as magnetic resonance imaging, genetics, and molecular biology. The most “historical” classification, endorsed by the World Health Organization (WHO), was based primarily on morphological and functional criteria, such as dilation, hypertrophy, restriction, and arrhythmia, without taking genetic factors into account [9].

Subsequently, scientific societies have proposed more nuanced classifications. The European Society of Cardiology (ESC) distinguishes familial/genetic forms from non-familial forms [10], whereas the American Heart Association (AHA) also encompasses primary genetic heart diseases [11]. A further step forward was represented by the MOGE(S) system, which introduces a multidimensional and “TNM-like” classification. In fact, it codifies cardiomyopathies through five domains: Morphology/phenotype (e.g., DCM, HCM, RCM, ACM, LVNC), Organ involvement (extracardiac involvement), Genetics (inheritance pattern), Etiology (genetic or acquired causes) and Functional stage (using ACC/AHA stages A-D and NYHA classes, American College of Cardiology) [12,13].

A major advancement, however, has been introduced with the more recent “Padua Classification”. Grounded in pathobiological principles, including genetics, molecular biology, histology, and clinical-morphological (morpho-functional) features revealed by imaging, particularly cardiac magnetic resonance (CMR), this system categorizes cardiomyopathies into three broad groups: hypertrophic/restrictive, dilated/hypokinetic, and cicatricial/arrhythmogenic, each with genetic or non-genetic variants [14,15].

This integrated approach, which correlates the clinical and morphological phenotype with molecular biology, enhances diagnostic accuracy and supports disease-specific management, facilitating not only more precise diagnosis but also the identification of targeted therapeutic interventions and personalized prognostic strategies.

Table 1 compares the classifications of cardiomyopathies, highlighting advantages.

## 3. Specific Cardiomyopathies: Morphological, Genetic and Pathogenetic Framework

### 3.1. Dilated Cardiomyopathy (DCM)

DCM manifests macroscopically with dilation of the ventricular chambers and reduced systolic function, most frequently affecting the left ventricle, and microscopically shows varying degrees of hypertrophic or atrophic cardiomyocytes, interstitial and/or replacement fibrosis, and sometimes inflammatory infiltration depending on the underlying etiology. These structural alterations are related to the clinical picture of heart failure, arrhythmias, and risk of SCD typical of DCM [16,17].

DCM is heterogeneous and involves genes encoding proteins of the sarcomere, cytoskeleton, nuclear lamina and, in some cases, components of the intercalated disc/desmosome. Among the most frequently implicated loci are sarcomeric genes such as *TTN* and *TNNT2*, cytoskeletal and mechanical transmission genes such as *DMD* and *FLNC*, and *LMNA*, which encodes nuclear lamin A/C. Furthermore, it has been recognized that variants in desmosomal genes can manifest clinically as dilatory phenotypes in some patients initially diagnosed with DCM. This plurality of molecular targets reflects the multifaceted nature of the disease [18,19,20].

Pathogenic mechanisms underlying DCM include: (1) sarcomeric dysfunction, in which mutations in contractile proteins alter contractile force and calcium sensitivity, predisposing to progressive failure; (2) nuclear instability and cell nuclear dysfunction induced by mutations in *LMNA* and lamina-associated proteins, resulting in impaired mechanical transmission, transcriptional regulation and increased oxidative stress; and (3) perturbations of protein homeostasis and cellular stress, including endoplasmic reticulum stress, altered mitochondrial proteostasis, and regulated cell death pathways such as apoptosis, ferroptosis, and autophagy dysfunction, which accelerate cardiomyocyte loss and fibrosis. These processes are not mutually exclusive but often interact, amplifying myocardial damage and leading to ventricular dilation and loss of contractile function [21,22,23].

The most recent evidence also highlights that the same genetic variant can have highly variable expressivity and penetrance, probably modulated by genetic modifiers, environmental stresses such as infections, toxins, alcohol abuse, and inflammatory processes. Consequently, a portion of DCM cases represent the final expression of a “multi-hit” attack on a predisposing genetic basis. This pathogenetic complexity motivates the modern diagnostic approach that integrates clinical data, imaging, histology, and genetic testing to stratify risk and guide precision therapies [24,25].

Table 2 summarizes the main genes associated with DCM and clinical implications, based on the recent literature [26,27,28,29,30,31].

### 3.2. Hypertrophic Cardiomyopathy (HCM)

HCM is characterized by well-defined histological features such as cardiac muscle cell disarray and extensive interstitial fibrosis. These structural alterations are not only peculiar but represent pathogenic substrates for arrhythmogenicity and the risk of SCD [32,33]. In particular, a recent study using fractional anisotropy magnetic resonance imaging (DT-CMR) demonstrated that even carriers of sarcomeric variants prior to hypertrophy (SARC+ LVH−) exhibit disarray microstructures, and that fibrosis correlates with prolonged ECG repolarization, suggesting a strong connection between microstructural changes, fibrosis, and electrical risk [32].

Genetically, mutations in sarcomere proteins are the most common cause of HCM. The genes most frequently involved are *MYH7* (β-myosin heavy chain) and *MYBPC3* (myosin C protein), which together account for about half of genetic cases. Other less common genes include troponins (*TNNT2*, *TNNI3*), tropomyosin (*TPM1*), and myosin light chain (*MYL3*) [33,34,35,36].

Regarding the genotype–phenotype correlation, there are conflicting data. Some studies suggested that mutations in *MYH7* were associated with earlier onset and more severe phenotypes, while those in *MYBPC3* had lower penetrance and manifested later [37]. However, a multicenter CMR analysis of over 350 subjects showed that no significant differences in morphology (wall thickness, ventricular masses, percentage of late gadolinium enhancement) emerged between patients with *MYH7* and *MYBPC3* mutations [38].

From a molecular perspective, allelic expression studies have highlighted that truncating mutations in *MYBPC3* do not always behave according to the classic haploinsufficiency model. In some patients, transcription of the mutated transcript is significantly lower than that of the wild type, but the abundance of the mutated protein can vary widely, suggesting that the stability of the mutated protein and its incorporation into the sarcomere may differ greatly depending on the type of mutation [39].

Furthermore, sarcomeric mutations are correlated with a greater extent of myocardial fibrosis compared to patients without mutations [40]. These data confirm that the genotype influences not only hypertrophy, but also other pathological aspects such as fibrosis, which in turn is an important arrhythmic risk factor.

Finally, the recent proposal of “gene-echocardiography” highlights that different patterns of hypertrophy, such as mid-septal thickening and septum-to-posterior wall ratio, are associated with specific genetic variants: *MYBPC3* mutants show a prevalence of mid-septal hypertrophy, while *MYH7* mutants tend to have higher septum/posterior wall ratio [41]. This close association between genetic variants and echocardiographic morphology could allow for more personalized diagnostics and more targeted genetic counseling for patients with HCM.

Table 3 summarizes the main genes associated with HCM and clinical implications, based on the recent literature [32,33,34,35,36,37,38,39,40,41].

### 3.3. Restrictive Cardiomyopathy (RCM)

RCM is characterized by marked myocardial stiffness, leading to diastolic dysfunction with severely impaired ventricular filling, while maintaining relatively normal ventricular dimensions and preserved systolic function. Significant left atrial or biatrial enlargement is often found, resulting from elevated ventricular filling pressure [42,43].

Genetically, RCM shows an important overlap with HCM, in fact, pathogenic variants in the same sarcomeric genes typical of HCM have also been identified in patients with RCM [43,44]. This suggests that, at least in some familial forms, RCM may not be a completely distinct disease, but rather a phenotypic end of the continuum of sarcomeric cardiomyopathies [14,43].

Mutations implicated in RCM include those affecting sarcomere proteins, such as troponins (*TNNI3*, *TNNT2*), myosin (*MYH7*), and myosin-binding protein C (*MYBPC3*), as well as cytoskeletal proteins such as desmin (*DES*) and filamin C (*FLNC*) [45,46,47]. Pathogenetically, these mutations can alter calcium sensitivity, promote the formation of protein aggregates, or disrupt the structural organization of sarcomeres and the cytoskeleton, resulting in increased myocardial stiffness [46].

The combination of these molecular factors, such as sarcomeric dysfunction, cytoskeletal protein aggregation and alterations in cellular homeostasis, contributes to the pathophysiology of RCM, characterized by impaired diastole, elevated filling pressure, and progressive heart failure [46].

Table 4 summarizes the main genes associated with RCM and clinical implications, based on the recent literature [42,43,44,45,46,47].

### 3.4. Arrhythmogenic Cardiomyopathy (ACM)

ACM is characterized by progressive fibrofatty replacement of the myocardium, initially prevalent in the right ventricle, but frequently extending to the left ventricle or in a biventricular form. This process causes dilation, dyskinesia, and aneurysm formation, creating a highly arrhythmogenic structural substrate. Fibrofatty infiltration and ventricular remodeling are well-documented by both advanced imaging and histopathological studies [48,49].

Diagnostically, the new Task Force 2023 criteria have improved sensitivity and specificity thanks to the integration of biventricular phenotypes, genetic markers and parametric imaging [49].

ACM has a strong genetic component, mainly associated with mutations in desmosome genes (*PKP2*, *DSP*, *DSG2*, *DSC2*, *JUP*). Mutations in *PKP2* represent the most frequent cause in Western cohorts, while variants in *DSP* and *DSG2* are associated with more severe phenotypes, often with marked left ventricular involvement and increased arrhythmic risk [50].

Recent cases have highlighted the pathogenic role of new variants, such as the *DSP* mutation p.K1165Rfs8*, associated with autosomal dominant phenotypes with sudden death at young age [51]. Further studies have shown how variants in *DSG2* can determine severe phenotypes already in pediatric age, with severe presentations [52].

Molecular features of ACM include mechanical disconnection of the intercalated disc due to the degradation of desmosomal complexes. Mutations such as those in *PKP2* increase vulnerability to mechanical stress and promote proteasomal degradation of desmosomal components [53]. Recent studies have also identified an involvement of the αVβ6/TGF-β pathway, which amplifies fibrotic remodeling [54].

An important step in the pathogenesis of ACM is the suppression of the Wnt/β-catenin pathway, mediated by the nuclear translocation of plakoglobin, which favors the activation of adipogenic programs [55]. In parallel, activation of the Hippo-YAP pathway, documented in PKP2-deficient cardiomyocytes, promotes adipogenesis, apoptosis, and a reduction in contractile mass [56].

Furthermore, evidence has shown that cardiac mesenchymal stromal cells (C-MSCs) also participate in the adipogenic phenotype in ACM; in fact, in affected patients, these cells show a marked tendency towards adipose differentiation [57].

Finally, novel integrated pathways and multimodal approaches to phenotype are described, broadening the understanding of the genetic, inflammatory and structural mechanisms underlying ACM [58,59].

Table 5 summarizes the main genes associated with ACM and clinical implications, based on the recent literature [48,49,50,51,52,53,54,55,56,57,58,59].

### 3.5. Left Ventricular Non-Compaction (LVNC)

LVNC is characterized by prominent trabeculations and deep intertrabecular recesses, with a bilaminar myocardial structure consisting of an endocardial non-compacted (spongious) layer and an epicardial compact layer [60,61].

However, diagnostic criteria are not univocal and numerous echocardiographic and CMR parameters exist, but the lack of a shared standard leads to a significant risk of overdiagnosis, especially because hypertrabeculation may be present in physiological conditions, such as athletes and pregnancy, or acquired [62].

Embryologically, it is hypothesized that LVNC results from an interruption of the normal process of myocardial compaction during fetal development [61,63]. However, more recently, it has been proposed that the compact and trabecular layers develop independently (“allometric growth”), partly overturning the classical idea of compaction block [61].

Genetically, LVNC is highly heterogeneous. Mutations in sarcomere genes, such as *MYH7*, *ACTC1*, *MYBPC3*, are frequently found, but defects in genes of cytoarchitecture and regulation of cardiac development have also been identified [64,65,66]. A large-scale study showed extensive genetic overlap with DCM and HCM, suggesting that for many patients, LVNC is not an independent cardiomyopathy but rather a phenotypic variant of other forms of cardiomyopathy [67].

Clinical and genetic overlap with DCM and HCM is well-documented, in particular, some carriers of genetic variants typical of DCM or HCM also fulfill the morphological criteria for LVNC, while other patients diagnosed with LVNC may present with typical features of HCM or DCM at the same or different stages of the disease [65,66]. This phenotypic continuum makes diagnosis and nosological definition particularly complex and raises doubts about its classification as a distinct cardiomyopathy.

In light of current genetic and clinical evidence, LVNC should be regarded as a heterogeneous condition rather than as a single nosological entity [60,61,62]. In a subset of patients, LVNC likely reflects a primary developmental cardiomyopathy; however, in many cases, it represents a morphological trait or phenotypic variant shared with HCM or DCM [63,64,65]. The extensive genetic overlap with sarcomeric and cytoskeletal cardiomyopathies, together with the frequent coexistence of morphological criteria for LVNC in patients with HCM or DCM, further supports this interpretation [66,67].

Recognizing LVNC as part of a broader phenotypic continuum has important clinical implications, as it helps to avoid overdiagnosis based solely on imaging criteria and promotes a comprehensive evaluation integrating genetic findings, family history, and functional assessment [60,61,62,63,64,65,66,67].

Table 6 summarizes the main genes associated with LVNC and clinical implications, based on the recent literature [60,61,62,63,64,65,66,67].

### 3.6. Integrative Molecular Mechanisms Across Cardiomyopathy Phenotypes

Although DCM, HCM, RCM, ACM, and LVNC are traditionally considered distinct entities, a growing body of molecular evidence supports the concept of a pathobiological continuum [45,46,47,48]. Shared pathogenic mechanisms include sarcomeric dysfunction, cytoskeletal instability, impaired proteostasis and maladaptive activation of signaling pathways involved in development and cellular stress responses [49,50,51,52]. Variants in sarcomeric genes, for example, may result in hypertrophic, dilated or restrictive phenotypes depending on their impact on cardiomyocyte contractile force, calcium sensitivity and energetic balance [53,54,55,56]. Similarly, defects in cytoskeletal and intercalated disc proteins compromise mechanical integrity and mechano-transduction, predisposing to ventricular dilatation or the formation of arrhythmogenic substrates [57,58,59].

In addition to structural alterations, dysregulation of intracellular signaling pathways plays a crucial role in phenotypic divergence [60]. Suppression of the canonical Wnt/β-catenin pathway and activation of the Hippo-YAP pathway promote adipogenesis and fibrosis in ACM [61,62,63], whereas excessive activation of TGF-β signaling contributes to fibrotic remodeling across several cardiomyopathy subtypes [64,65]. Alterations in proteostasis, endoplasmic reticulum stress and autophagy dysfunction further exacerbate cardiomyocyte loss and myocardial remodeling [66,67].

Taken together, these shared and divergent molecular mechanisms provide a unifying framework linking myocardial morphology, genomic alterations, and clinical phenotypes, supporting the concept that cardiomyopathies represent overlapping expressions of interconnected biological processes rather than entirely separate diseases [45,46,47,48,49,50,51,52,53,54,55,56,57,58,59,60,61,62,63,64,65,66,67].

## 4. Advanced Diagnostic Techniques

### 4.1. Genetic Analysis

In recent years, the use of NGS techniques has revolutionized the genetic approach to cardiomyopathies, allowing for the analysis of target gene panels, the WES, or even the WGS in clinical practice. Several studies are described in the literature [68,69,70].

A study of patients with inherited cardiomyopathies used a personalized NGS panel of over 200 genes, finding a high percentage of variants even in “uncommon” genes not yet strongly associated with the disease [68].

In an analysis of 260 patients with suspected inherited heart disorders, the use of WES increased the diagnostic rate up to 11.7% compared with targeted panels and showed an increase in variants of uncertain significance (VUSs) [69].

In an Italian cohort of pediatric and adult patients with cardiomyopathies, the use of WES on in silico panels allowed for the identification of a molecular diagnosis in 21.5% of cases, with a higher yield in familial cases [70].

A frequent problem in genetic interpretation is caused by VUSs. In a reanalysis study of patients with HCM, DCM, and ACM, approximately 95% of identified VUSs remained so after 3–5 years, although a small percentage were reclassified as pathogenic or benign. These reclassifications are essential to improve diagnostic accuracy and to guide family follow-up [71].

It is increasingly evident that genetic testing results exert a direct influence on clinical decision-making, particularly in inherited cardiomyopathies and arrhythmogenic disorders [45,46,47,48,49,50]. The identification of pathogenic variants in high-risk genes, such as *LMNA*, *DSP*, or *FLNC*, has been consistently associated with an increased risk of malignant ventricular arrhythmias and may prompt earlier consideration of implantable cardioverter defibrillators (ICDs), even in individuals with only mild systolic dysfunction [51,52,53,54,55,56,57,58]. Moreover, genetic findings inform cascade family screening and facilitate the development of personalized surveillance strategies for genotype-positive/phenotype-negative individuals [59,60,61,62,63,64,65,66]. Consequently, genetic testing constitutes not merely a diagnostic tool, but a cornerstone for risk stratification and personalized preventive care in contemporary clinical practice [67,68,69,70,71,72].

Furthermore, genetic counseling, given the complexity of the results (pathogenic variants, VUS, negative results), is essential to allow patients to receive pre-test and post-test counseling conducted by an experienced geneticist or genetic counselor, to understand the potential clinical, psychological, and family implications [72]. The intervention of a genetic counselor allows us to discuss the opportunity for screening in other family members, to explain the limitations of the test, and to plan the periodic review of the variants based on new evidence.

### 4.2. Molecular Pathology

In cardiomyopathy studies, immunohistochemistry remains a mainstay for the molecular analysis of myocardial tissue. Using specific antibodies in biopsies or autopsies, it is possible to detect the distribution and aberrant expression of key proteins, such as desmosomal proteins, extracellular matrix proteins, and stress factors, providing histological information that complements genetics.

In parallel, omics techniques, such as transcriptomics and proteomics, are becoming increasingly central to cardiomyopathy research. A recent study performed a proteomic profile on human myocardium in HCM, identifying over 7000 proteins and demonstrating significant alterations in signaling pathways, such as MAPK, ubiquitin-mediated, Hippo, confirmed by transcriptional analyses [73]. Furthermore, single-cell transcriptomics (single-cell RNA-Seq) has revealed specific changes in the cardiac fibroblast population in DCM models, offering new insights into how inflammation and cellular remodeling contribute to pathogenesis [74].

Digital pathology and artificial intelligence (AI) are also emerging strongly in cardiac pathology; in fact, machine learning and deep learning algorithms are used to analyze digitized histological images, recognizing subtle and potentially predictive patterns of cardiomyopathies [75,76,77]. In a clinical setting, these technologies could integrate histological, genetic, and clinical data to improve diagnosis, stratify risk, and guide therapy in a more objective and personalized manner [78].

### 4.3. Genotype–Phenotype Correlations

Genotype–phenotype correlations in cardiomyopathies have important implications for prognosis. A study on a cohort of 281 patients with inherited cardiomyopathies (DCM, ACM, biventricular forms) showed that classification based on genotype more accurately predicts the risk of SCD or ventricular arrhythmias than a classification based on phenotype alone. Variants in *LMNA*, *DSP*, *PKP2* and *FLNC* were found to be associated with a significantly higher risk of severe arrhythmic events [79].

In DCM, analysis of a multicenter cohort demonstrated that specific genes present phenotypes; carriers of variants in *FLNC*, *LMNA*, *DSP* and *PLN* showed a higher arrhythmic risk, while mutations in *BAG3*, *TNNT2*, *DMD* and *TTN* are related to increased ventricular volumes and ejection fraction dysfunction [80]. A meta-analysis of over 8000 patients confirmed that *LMNA* and *PLN* are associated with an increased incidence of sudden death, cardiac transplantation, or ventricular arrhythmias compared with sarcomeric genes [81].

For HCM, recent studies show that patients with pathogenic or probable pathogenic (P/LP) variants tend to present earlier diagnosis and more marked hypertrophy than patients without identifiable mutations [82]. In a specialized center, stratification of HCM patients by genotype revealed that the “genotype-positive” group had a significantly higher composite risk (including heart failure, arrhythmias, interventions) than the “genotype-negative” one [83].

In ACM, genotype–phenotype correlation is crucial for clinical management, in particular, *PKP2* is frequently associated with forms with right ventricular involvement and arrhythmias, while *DSP* is often related to subepicardial left ventricular scarring and may present with near-normal ECG [84]. Preclinical models have in fact helped to clarify how these variants influence the structure and electrical behavior of the myocardium [85].

In pediatric patients with inherited cardiomyopathy, variants in *MYH7* and *MYBPC3* have been associated not only with earlier clinical manifestations but also with worse prognosis, with an increased need for implantable cardioverter defibrillators or cardiac transplantation [86].

Genotype–phenotype correlations not only clarify shared pathogenic mechanisms but also offer a powerful prognostic tool: identifying the causal gene can guide risk stratification, surveillance, and personalized therapy.

## 5. Sudden Cardiac Death and Molecular Autopsy

SCD represents a complex condition that demands an integrated diagnostic strategy due to its remarkable etiological heterogeneity and the need for a systematic investigation to elucidate its multiple causes. The literature underscores how traditional autopsy, combined with histological and immunohistochemical examination, remains the cornerstone for identifying the structural causes of SCD and distinguishing primary myocardial alterations from secondary conditions such as ischemia or toxicity [7,87,88,89,90,91].

Nevertheless, a substantial proportion of SCD cases remain unexplained after conventional autopsy, leading to a classification as sudden arrhythmic death syndrome (SADS). In such instances, MA, employing dedicated NGS genetic panels or broader approaches such as WES, enables the detection of molecular defects that may not be apparent at histopathology alone. This approach has proven invaluable in revealing pathogenic variants in genes linked to inherited arrhythmogenic conditions [92,93,94,95,96,97].

Furthermore, MA has important clinical and familial implications: identifying a pathogenic mutation in the decedent allows clinical screening and genetic testing for first-degree relatives, facilitating the early detection of individuals at risk of inherited cardiomyopathy or arrhythmic disease. This is especially pertinent in autosomal-dominant disorders [98,99,100,101,102,103].

From a medico-legal standpoint, combining conventional pathology with MA helps to reduce interpretative errors and ascertain the cause of death more precisely, thus supporting more robust judicial and insurance outcomes [104,105,106].

In addition, recent studies further support the value of MA. NGS-based analysis postmortem has been shown to substantially increase diagnostic yield in unexplained SCD cases, identifying clinically actionable variants in a significant percentage of subjects [104,107]. A phenotype-driven approach to MA in a large cohort uncovered likely pathogenic variants in ion-channel and cardiomyopathy genes, with subsequent family screening uptake significantly higher when a molecular diagnosis was made [108]. Moreover, in a registry of athlete SCD cases, MA yielded clinically actionable variants in 17% of decedents, emphasizing its utility even in seemingly healthy individuals [109].

Despite its substantial clinical and diagnostic value, MA is subject to several practical limitations [87,88,89]. DNA degradation in post-mortem samples, variability in the genetic panels employed across different institutions, and the frequent identification of VUSs can complicate the interpretation of results [90,91,92,93,94,95].

Furthermore, the ethical implications associated with communicating genetic findings to family members necessitate careful management, structured genetic counselling, and the involvement of a multidisciplinary team [96,97,98,99,100,101,102] including cardiovascular pathologists, clinical geneticists, cardiologists, forensic experts, and imaging specialists. This collaborative framework enables more accurate diagnoses and establishes preventive and monitoring strategies for relatives, thereby translating molecular findings into concrete clinical benefits.

## 6. Clinical Implications and Targeted Therapies

The integration of genetic information into clinical practice has profoundly transformed the management of inherited cardiomyopathies, paving the way for a precision medicine approach that extends beyond conventional diagnosis and risk stratification. An improved understanding of the specific molecular mechanisms associated with distinct genotypes now enables the identification of actionable therapeutic targets and the development of innovative, mechanism-based treatment strategies [110,111].

In recent years, gene-based therapies and genome editing approaches have attracted considerable interest. Preclinical studies have demonstrated that CRISPR/Cas9-based technologies, including base editing and prime editing, can correct pathogenic variants in sarcomeric genes such as *TNNT2* and *MYH7* in cellular models derived from induced pluripotent stem cells (iPSCs), resulting in the restoration of contractile function and normalization of sarcomeric architecture [112,113]. Similarly, murine models of HCM have shown significant attenuation of myocardial hypertrophy and fibrosis following targeted genetic correction [113].

However, the clinical translation of these technologies remains challenging. Major limitations include the efficiency and specificity of cardiac delivery of viral vectors, particularly adeno-associated viruses (AAVs), the risk of off-target genomic effects, the immunogenicity of both vectors and genome-editing components, and the ethical and regulatory complexities associated with permanent genomic modifications [111,114,115].

In parallel, alternative or complementary therapeutic strategies are emerging as more readily applicable options. In this context, the use of small molecules represents a form of *functional precision* or *semi-precision* medicine. A paradigmatic example is provided by selective cardiac myosin inhibitors, such as mavacamten and aficamten, in HCM. These agents act by directly modulating sarcomeric contractility and have demonstrated clinical efficacy in improving symptoms, reducing left ventricular outflow tract obstruction, and promoting reverse myocardial remodeling [111,114].

Additional approaches include the use of antisense oligonucleotides and RNA interference technologies to selectively downregulate dominant mutant alleles, as well as the modulation of secondary signaling pathways involved in pathological cardiac remodeling, including the TGF-β, Wnt/β-catenin, and Hippo-YAP pathways. These pathways have been implicated in myocardial fibrosis and adipogenesis, particularly in ACM [54,55,56,115].

Finally, emerging evidence suggests that, especially in HCM, alterations in coronary microcirculation and the regulation of vascular tone may represent additional therapeutic targets, further broadening the scope for integrated treatment strategies capable of modifying disease progression beyond symptomatic control [116].

Although RNA-based therapies and genome-editing technologies offer highly promising prospects for the treatment of inherited cardiomyopathies, their clinical application is currently limited by several unresolved challenges, including efficient and tissue-specific cardiac delivery, potential off-target effects, and uncertainties regarding long-term safety [108,109,110,111]. Most of the gene therapy and genome-editing strategies remain in experimental or preclinical stages, underscoring the need for cautious evaluation of their true translational potential and clinical scalability [112,113,114]. At present, targeted pharmacological interventions guided by molecular and genetic profiling represent the most concrete and readily applicable form of precision medicine in inherited cardiomyopathies [115,116].

## 7. Current Challenges and Future Prospects

Current challenges in the management of inherited cardiomyopathies include the marked phenotypic variability, which makes it difficult to predict clinical evolution even in patients with the same genetic variant, as highlighted by studies showing how polygenes and common modifiers influence disease penetrance and expressivity [117].

The interpretation of genetic variants, especially VUSs, remains critical. In fact, current guidelines are heterogeneous and often require periodic re-evaluations, given that the classification criteria (ACMG/AMP) depend strongly on the clinical and family context [118,119].

An additional challenge is the inconsistent clinical guidelines, which in many cases do not fully reflect the molecular and genetic complexity of cardiomyopathies, raising the need for shared and updated standards [119]. Furthermore, there is an urgent need for well-maintained international biobanks and databases to enable the systematic collection of large-scale phenotypic, genetic, and multi-omics data and foster collaborative research [120,121].

The future prospects are promising. Multi-omics integration (genomics, transcriptomics, proteomics, metabolomics) could provide a deeper understanding of pathogenic mechanisms and improve risk stratification [73]. In terms of precision medicine, the combination of genetic information with molecular and clinical data will allow for personalized therapy based on the individual’s genotype and biological profile [111]. Innovative biomarkers, including those derived from RNA, proteins, and metabolites, could be used to monitor disease and assess therapeutic response [111,122,123,124].

At the same time, AI-based predictive software, which analyzes clinical, genetic, and imaging data, represents a powerful resource for building accurate predictive models [75,78].

Finally, new molecular discoveries could promote a revision of the classification of cardiomyopathies, moving from a phenotypic classification to one based on molecular mechanisms, more in line with biological and therapeutic reality [79].

## 8. Conclusions

Cardiomyopathies are a heterogeneous group of pathologies in which the integration of morphological, genetic, and molecular data has become essential for accurate diagnosis, correct prognostic stratification, and the development of personalized therapeutic pathways. A review of available evidence shows how a multidisciplinary approach, involving cardiologists, pathologists, geneticists, bioinformaticians, and imaging specialists, is essential to correctly interpret the complexity of phenotypes and the impact of genetic variants, helping to reduce the number of incomplete or inconclusive diagnoses.

Current challenges, including phenotypic variability, interpretation of variants, the need for more robust data, and the lack of uniform guidelines, point the way for future developments. The expansion of biobanks, the construction of harmonized international databases, and the adoption of integrated multi-omics approaches will be essential to better understand pathogenic mechanisms and identify new disease biomarkers. At the same time, advances in data analytics and AI technologies will support the creation of increasingly accurate predictive models, contributing to truly precision medicine.

In conclusion, the combination of technological innovation, multidisciplinary collaboration, and advanced molecular approaches represents the key to improving diagnosis, clinical management, and therapeutic options for patients with cardiomyopathies, paving the way for a future in which classifications and interventions will be increasingly based on deep and shared biological foundations.

## Figures and Tables

**Table 1:** Evolution of cardiomyopathy classification: a comparison between historical and modern systems.

Classification System	Main Features	Advantages
**WHO/** **ISFC**	Classification based on morphology and function: DCM, HCM, RCM, ARVC, Unclassified; distinguishes primitive and secondary forms.	•Simple and easy to apply clinically •First international standardization •Useful for describing macroanatomical phenotypes
**AHA**	Introduces the distinction between genetic and acquired cardiomyopathies; recognizes: HCM, DCM, RCM, ARVC, LVNC.	•First systematic integration of genetics •Improved support for family counseling •Recognizes LVNC as a distinct entity
**ESC**	Classification based on phenotype and division into familial/genetic and non-familial/non-genetic forms.	•More clinical and practice-oriented approach •Incorporates family history as a diagnostic criterion •Recognizes mixed phenotypes and secondary forms
**MOGE(S)**	Based on a TNM-like system: M (morphology), O (organ involvement), G (genetics), E (etiology), S (functional stage).	•Extremely personalized and comprehensive approach •Excellent description of complex phenotypes •Includes genetics and etiology in a structured manner
**Padua**	New integrated classification based on pathobiology: (1) Hypertrophic/restrictive (2) Dilated/hypokinetic (3) Cicatricial/arrhythmogenic It distinguishes between genetic and non-genetic, integrating imaging, genetics, and histology.	•First classification to integrate genetics, histology, imaging, and clinical features •Identifies biological patterns with prognostic relevance •Adapted to the era of precision medicine

Data summarized from references [9,10,11,12,13,14,15].

**Table 2:** Genes associated with DCM and clinical implications.

Gene	Cell Function/Structure	Clinical Implications
***TTN***	Titin (sarcomeric)	Often associated with systolic dysfunction; in some cases, good recovery with cardiac therapy.
***LMNA***	Lamin A/C (nuclear lamina proteins)	Mutations associated with elevated risk of ventricular arrhythmias, conduction system disease, and worse prognosis with advanced heart failure.
***FLNC***	Filamin C (cytoskeletal protein)	Often associated with an arrhythmogenic phenotype, with an increased risk of sudden death or advanced ventricular arrhythmias.
***BAG3***	Co-chaperone protein	Mutations associated with advanced heart failure and poor clinical prognosis.
***DES***	Desmin (intermediate cytoskeletal protein)	Mutations can cause cytoskeletal disorganization, cardiac weakness, and potential arrhythmia.
***DSP***	Desmoplakin (intercellular junctions)	It may be associated with phenotypes with junctional involvement, arrhythmias and possible overlap with ACM.
***RBM20***	RNA-binding protein	Mutations are associated with an aggressive form of DCM, with arrhythmias and a rapid progression; generally worse prognosis.
***SCN5A***	Cardiac sodium channel Nav1.5	Mutations can cause arrhythmias, electrical dysfunction, and increased risk of sudden death.
***TNNT2***	Troponin T	Mutations can alter contractile regulation, leading to systolic dysfunction and heart failure.
***TNNC1***	Troponin C	Alterations may affect calcium sensitivity and sarcomere mechanics.
***MYH7***	β-Myosin (sarcomeric)	Associated with both DCM and other cardiomyopathies, it may be involved in contractile dysfunction and cardiac remodeling.

Data summarized from references [16,17,18,19,20,21,22,23,24,25,26,27,28,29,30,31].

**Table 3:** Genes associated with HCM and clinical implications.

Gene	Cell Function/Structure	Clinical Implications
***MYH7***	β-Myosin heavy chain (sarcomere)	• Earlier onset, • More marked hypertrophy, often septal, • Greater risk of arrhythmia and fibrosis compared to MYBPC3.
***MYBPC3***	Myosin-binding protein C (sarcomere)	• Later onset, • Variable penetrance, • Extensive fibrosis in mutant cases, • Protein variability (model beyond simple haploinsufficiency).
***TNNT2***	Cardiac troponin T	• Mild hypertrophy with disproportionate arrhythmic risk, • Increased risk of sudden death.
***TNNI3***	Cardiac troponin I	• Wide phenotypic variability, • Hypertrophy pattern not always severe but increased arrhythmic risk.
***TPM1***	Tropomyosin α	• Variable hypertrophy, • Possible association with early onset familial forms.
***ACTC1***	Cardiac actin	• Mild-moderate hypertrophy, • Marked familial variability.
***MYL2/MYL3***	Myosin regulatory/essential light chain	• Septal hypertrophy pattern, • Possible increased incidence of arrhythmias.
***FLNC***	Filamin C (cytoskeleton)	• HCM–ACM hybrid phenotypes, • Ventricular arrhythmias and marked fibrosis.
***PLN***	Phospholamban (calcium homeostasis)	• Mixed HCM/DCM phenotypes, • Early systolic dysfunction.

Data summarized from references [32,33,34,35,36,37,38,39,40,41].

**Table 4:** Genes associated with RCM and clinical implications.

Gene	Cell Function/Structure	Clinical Implications
***TNNI3***	Troponin I (sarcomeric)	Increased sensitivity to Ca^2+^, myocardial stiffness, typical RCM phenotype; frequent arrhythmias.
***TNNT2***	Troponin T (sarcomeric)	Severely impaired diastole, possible mixed HCM/RCM forms.
***MYH7***	β-Myosin heavy chain	Phenotype overlaps with HCM; variable penetrance; mild hypertrophy and rigidity.
***MYBPC3***	Myosin-binding protein C	Altered diastolic pattern; may mimic restrictive HCM.
***ACTC1***	Cardiac actin	Elevated myocardial stiffness and altered thin filament dynamics.
***DES***	Desmin (cytoskeleton)	Presence of inclusion bodies, structural dysfunction; often associated myopathy.
***FLNC***	Filamina C	Mixed RCM/ACM phenotypes; high arrhythmic risk; marked fibrosis.
***LMNA***	Lamina A/C (nucleus)	Conduction system involvement, arrhythmias, ventricular stiffness.
***BAG3***	Protein co-chaperone	Proteostatic dysfunction; restrictive pattern with early heart failure.
***PLN***	Phospholamban (Ca^2+^ homeostasis)	Diastolic filling dysfunction, RCM/DCM hybrid phenotypes.

Data summarized from references [42,43,44,45,46,47].

**Table 5:** Genes associated with ACM and clinical implications.

Gene	Cell Function/Structure	Clinical Implications
***PKP2***	Plakophilin-2 (desmosome)	Typical right ventricular involvement; high risk of ventricular arrhythmias; early fibroadipose substrate.
***DSP***	Desmoplakin	Severe phenotypes, often with left ventricular involvement; greater extent of fibrosis; major arrhythmic events.
***DSG2***	Desmoglein-2	Biventricular fibrofat replacement; risk of ventricular tachycardia; marked structural progression.
***DSC2***	Desmocollina-2	Variable phenotypes, often biventricular; possible role as a biomarker (early remodeling).
***JUP***	Plakoglobin	Involved in nuclear translocation and Wnt suppression; severe phenotypes with dominant adipogenesis.
***FLNC***	Filamin-C (cytoskeleton)	ACM-like phenotypes with diffuse fibrosis; high arrhythmic risk; LV involvement.
***LMNA***	Lamina A/C (nucleus)	Marked electrical involvement; hybrid ACM/DCM phenotypes; high risk of sudden death.
***TGFB1*** **/integrin αVβ6 signaling**	Extracellular remodeling	Contributes to fibrosis through TGF-β pathway; tissue progression.
**Pathway Wnt/β-catenin**	Regulation of cell fate	Suppression of canonical Wnt → adipogenesis and myocardial fibrosis.
**Pathway Hippo-YAP**	Regulation of proliferation/apoptosis	Activation in PKP2 deficiency → adipogenesis, cardiomyocyte loss.

Data summarized from references [48,49,50,51,52,53,54,55,56,57,58,59].

**Table 6:** Genes associated with LVNC and clinical implications.

Gene	Cell Function/Structure	Clinical Implications
***MYH7***	β-Myosin heavy chain (sarcomere)	LVNC phenotype isolated or combined with HCM/DCM; risk of systolic dysfunction; variable penetrance.
***MYBPC3***	Myosin-binding protein C	LVNC with possible hypertrophy; frequent overlap with HCM; moderate arrhythmic risk.
***ACTC1***	Cardiac actin	Variable phenotypes: LVNC, HCM-like, DCM-like; contractile dysfunction.
***TNNT2***	Troponin T	High heterogeneity: familial LVNC, risk of arrhythmias, progression to LV dysfunction.
***TNNI3***	Troponin I	Intermediate LVNC–RCM phenotypes; increased myocardial stiffness.
***TAZ*** **(G4.5)**	Tafazzin (mitochondrial phospholipids)	Associated with Barth disease; LVNC with severe systolic dysfunction and metabolic myopathy.
***MIB1***	E3 ubiquitin ligase (Notch signaling)	Alterations in cardiac development; coexistence of LVNC with embryonic arrest anomalies.
***LMNA***	Nuclear lamina A/C	Hybrid LVNC/DCM phenotypes; high risk of ventricular arrhythmias; progressive dysfunction.
***DES***	Desmin (cytoskeleton)	LVNC with associated myopathy; increased arrhythmogenic burden.
***FLNC***	Filamina-C	LVNC with fibrosis, arrhythmias, and risk of major events; overlapping ACM/DCM phenotypes.
***HCN4***	Funny channel (pacemaker)	LVNC associated with bradycardia, sinus node dysfunction, dilated aorta.

Data summarized from references [60,61,62,63,64,65,66,67].

## Data Availability

No new data were created or analyzed in this study.
